# Practical Points

**Published:** 1896-01-25

**Authors:** 


### PRACTICAL POINTS.

[Questions are invited for this column on any point in the administration of hospitals and asylums which may prove of difficulty or interest to our readers. All communications should be marked "Practical Points," addressed to the Editor of The Hospital, 428, Strand, W.C.]

*Nurses' Homes.—A Hospital Matron, whose committee are considering the question of building a new home for their nursing staff, writes to ask the names of the most recently built and best constructed nursing homes, with a view to gaining therefrom hints for guidance in the matter of the new buildings.*

In reply, we may say that in our view anyone who has seen the Nurses' Home at the Edinburgh Royal Infirmary, and has studied it practically and understands clearly the point of view upon which the home has been constructed and managed, need not see anything else. A full account of this home appeared in The Hospital for Nov. 25th, 1893, p. 125. Other plans will also be found in The Hospital Nursing Supplement for October 6th, 1894, p. viii., with suggestive criticisms, the result of a novel competition giving the nurses' home from the point of view of the nurses themselves. The numbers containing these can be obtained from the Manager, 428, Strand. There is a good home at the London Fever Hospital, Islington, which is worth studying in connection with the Edinburgh Home, and a new home at Wigan, plans of which will shortly be published in The Hospital. The London Hospital Nursing Home and others in the metropolis might be worth visiting. Much depends upon the architect elected to prepare the plans, and more upon the person whom the committee call in to advise them as to the plans to approve before the buildings are begun.

